# Ocular symptoms secondary to meningeal carcinomatosis in a patient with lung adenocarcinoma: a case report

**DOI:** 10.1186/1471-2415-12-65

**Published:** 2012-12-18

**Authors:** Alfonso L Sabater, Luis M Sadaba, Elisa de Nova

**Affiliations:** 1 Department of Ophthalmology, Clínica Universidad de Navarra, Navarra, Apartado 4209, Pamplona, 31008, Spain

## Abstract

**Background:**

Meningeal carcinomatosis (MC) is a rare complication associated with hematologic and solid tumors. MC develops when malignant cells gain access to the leptomeningeal space, producing several clinical symptoms. Loss of vision and ocular motility deficit are the most frequent ocular symptoms reported. Fundus examination usually appears normal, although optic nerve alterations like optic atrophy or papilledema have been described. MC diagnosis is usually completed by magnetic resonance imaging (MRI) and cerebrospinal fluid (CSF) analysis. Indicated treatment for MC usually involves intrathecal chemotherapy combined with radiotherapy, although survival rate is extremely low.

**Case presentation:**

A 66-year old man with stage IV metastatic lung adenocarcinoma, presented to the Ophthalmology Department with a two-month history of double vision, soft headaches and dizziness episodes. The patient presented a best visual corrected acuity of 0.7 in his right eye and 0.8 in his left eye. Diplopia was corrected with 6-prism diopters base-out prism in right eye. Funduscopy showed a bilateral papilledema, juxtapapillary exudates and splinter hemorrhages. Brain MRI showed a diffuse leptomeningeal enhancement in cortical sulcus. Lumbar puncture was performed and cerebrospinal fluid (CSF) cytology revealed malignant cells compatible with a diagnosis of MC. Intrathecal chemotherapy was administered.

**Conclusion:**

MC is a serious complication of systemic cancer patients, involving a poor prognosis. Early diagnosis is extremely important, although treatment is frequently aimed to reduce the symptoms and extend survival. Eye symptoms may be the chief complaint, so MC should be considered in any patient with vision loss or diplopia accompanied by neurologic symptoms and in the absence of an intraocular cause, especially in the context of systemic cancer.

## Background

Meningeal carcinomatosis (MC) is a rare and severe complication of a number of neoplastic processes. Hematologic tumors are the most frequent cause of MC, particularly acute lymphoblastic leukemia and non-Hodking lymphoma. Among solid tumors, the most frequent causes of MC are breast and lung adenocarcinoma as well as melanoma. The incidence of this complication is around 3% in breast cancer, 7% in lung cancer and 1.5% in melanoma [1]. MC is the first manifestation of systemic cancer in only 5-10% of patients, although it is frequently seen in patients with disseminated systemic disease [2,3].

MC occurs when tumor cells reach the leptomeningeal space either through the blood, the cerebrospinal fluid (CSF) or by growing along nerve and vascular sheaths. As a consequence, most patients present with different multifocal neurological symptoms that may involve the cranial nerves (oculomotor, facial, cochlear and optic), the spine and the cerebrum [3,4]. Ocular symptoms were first reported by Katz, being diplopia and vision loss the most frequent ocular complaints [1,5].

Early diagnosis and treatment are usually very complicated. Magnetic resonance imaging (MRI) is more sensitive than computed tomography (CT) scan in detecting brain meningeal dissemination, although the spine should also be assessed by MRI or CT myelography [6]. MRI may show focal or diffuse leptomeningeal enhancement as well as tumoral lesions [7]. However, these findings are non-specific, and should be evaluated in the particular clinical context. On the other hand, CSF analysis should be performed in all patients with suspected MC. Malignant cells are detected in the CSF in only 50% of patients with MC in the first lumbar puncture [8]. Therefore, repeated CSF sampling is usually needed. Finally, the CSF protein concentration (50 mg/dl) and the opening CSF pressure (>25 cm H2O) are frequently elevated, whereas the glucose concentration (<60 mg/dl) is usually decreased.

Differential diagnosis should include inflammatory processes (rheumatoid arthritis, eosinophilic granuloma), infections, granulomatous infiltrations (sarcoid, tubercular), previous administration of chemotherapy, venous thrombosis, subarachnoid haemorrhage, trauma and previous intracranial surgeries [9].

MC is associated with a bad prognosis with a mean survival rate of 3–6 months [10]. Treatment aims to extend survival and stabilize or improve neurological symptoms [3]. Surgical treatment includes the placement of a ventriculoperitoneal shunt in patients with symptomatic hydrocephalus, although these patients have very poor prognosis [11,12]. Involved-field radiotherapy may also be applied to patients with MC, although it does not commonly achieve a prolonged survival [13]. On the other hand, intrathecal or ventricular administered chemotherapy is a more selective and less toxic treatment for patients with MC [8]. Methotrexate, cytosine arabinoside and thiotepa are the most frequent drugs, although other substances have been tested in several clinical trials [1].

## Case presentation

A 66-year old white man with a history of benign prostatic hyperplasia and tuberculosis, presented to the Ophthalmology Department complaining of double vision, non-intense headaches and dizziness episodes from two months ago. The patient was diagnosed fours month ago of stage IV lung adenocarcinoma with pericardial and bone metastases, in treatment with Alimta plus Carboplatin and Zoledronic Acid. Brain MRI performed four days ago showed no brain lesions.

**Figure 1:**
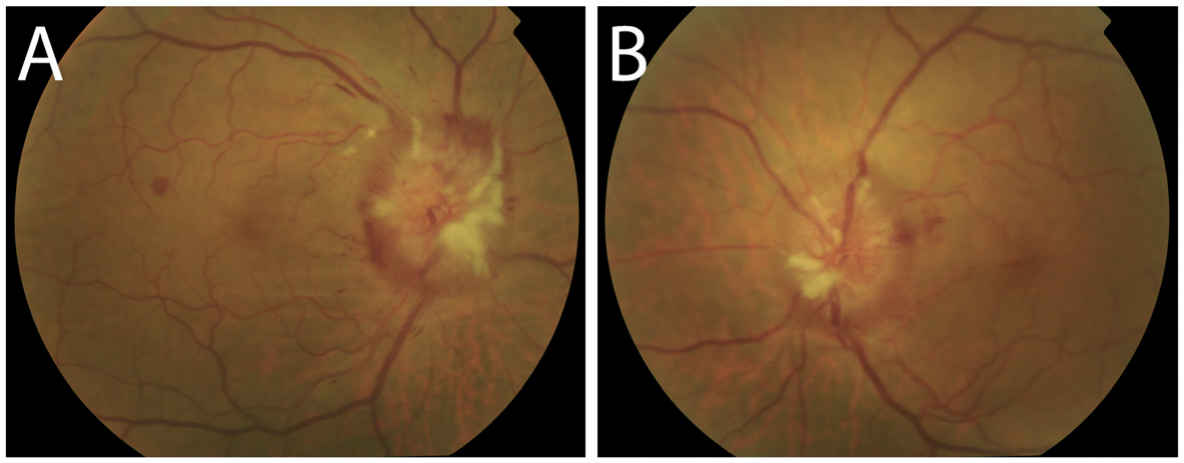
**Funduscopic examination. A**: Right eye. **B**: Left eye. Funduscopic examination revealed bilateral papilledema with juxtapapillary exudates and splinter hemorrhages. The macula and blood vessels showed no alterations.

In the ophthalmologic exploration the patient presented a best visual corrected acuity of 0.7 and 0.8 in right and left eye respectively, with diplopia corrected with 6-prism diopters base-out prism in right eye. Funduscopy showed a bilateral papilledema with juxtapapillary exudates and splinter hemorrhages (Figure 1), suggesting intracranial hypertension. The macula and blood vessels showed no alterations.

**Figure 2:**
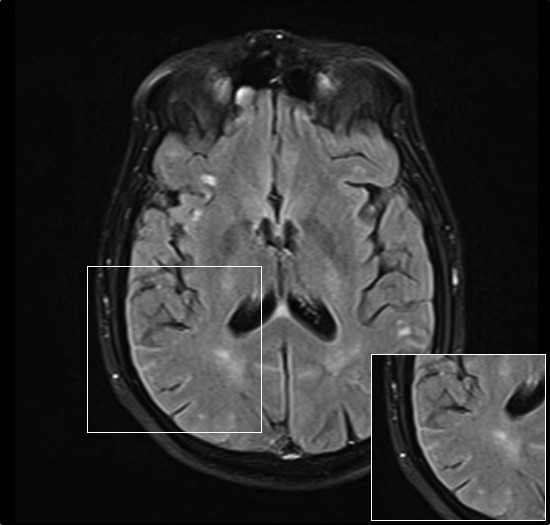
**Brain Magnetic Resonance Imaging.** Axial T2-FLAIR brain MRI scan shows leptomeningeal enhancement in right parietal cortex.

The patient was admitted for further evaluation. One week later the patient suffered two consecutive auto-limited partial motor crises. CT scan showed no intracranial hemorrhages or orbital lesions. In contrast, brain MRI showed a diffuse leptomeningeal enhancement in cortical sulcus, especially in frontal, temporal and parietal lobes (Figure 2). Lumbar puncture was performed and CSF was analyzed showing aggregates of atypical epithelial cells, lymphocytes, cell detritus and red blood cells. CSF flow analysis revealed a pressure of 40 cm H2O, a protein concentration of 68.30 mg/dl and a glucose concentration of 35 mg/dl. Based on clinical symptoms and according to the test results, the patient was diagnosed of MC. Next day, a 15 mg dose of intrathecal methotrexate was administered. Methotrexate was further administered on a weekly basis. Five weeks later, CSF was analyzed and tumor cytology was negative. Consequently, Methotrexate was administered every three weeks. Finally, after 1.5 months of treatment, the patient referred diplopia stabilization.

## Conclusion

MC is becoming a relatively frequent complication secondary to systemic cancer patients, as a consequence of better survival rates [8]. MC occurs when tumor cells disseminate and grow in the leptomeningeal space and/or the cerebrospinal fluid (CSF). Consequently, these patients may present with different multifocal neurological symptoms. As demonstrated in this case, our patient complained of double vision as well as headaches and dizziness episodes. Furthermore, funduscopy revealed an evident bilateral papilledema that in association with a metastatic lung adenocarcinoma clearly orientated the diagnosis towards a neurologic process. In these cases, early diagnosis is important, although treatment is intended to reduce the symptoms and at best extend survival, especially when neurologic symptoms are already present [14].

Currently, diagnostic approach to ascertain MC requires performing cranial and spinal MRI, CSF cytology, CSF flow analysis and CSF glucose and protein concentration [15]. In first place, a CT scan was performed and it showed no brain lesions. In contrast, brain MRI, which is more sensitive that CT for detecting brain meningeal dissemination, showed a diffuse leptomeningeal enhancement, which was compatible with the diagnosis of MC. On the other hand, spinal MRI was completely normal. In second place, CSF cytology showed several malignant aggregated cells in the sample. However, in some other cases where there is a clinically suspected MC and negative CSF cytology, different tumour-specific markers may be evaluated [11]. Finally, CSF flow analysis revealed an elevated intracranial pressure, a high protein concentration and a low glucose concentration. Accordingly, diagnosis of MC was made based on the clinical symptoms and the test results.

The patient received treatment for MC with intrathecal methotrexate, which was further administered on a weekly basis until CSF cytology became negative after five weeks. Then, treatment was administered every three weeks. Although systemic chemotherapy at high doses could be useful in this case, intrathecal chemotherapy allows a better distribution throughout the subarachnoid space with less systemic toxicity [1]. After 1.5 months of treatment, the patient referred stabilization of the neurological symptoms, including diplopia. Intrathecal methotrexate acts by inhibiting the metabolism of folic acid. Therefore, inhibits the synthesis of DNA, RNA, thymidylates, and proteins, having a greater toxic effect on rapidly dividing cells. Thus, methotrexate was effective in this case by reducing the uncontrolled local growth of tumoral cells that were responsible for diplopia and other neurological symptoms.

In conclusion, ocular symptom(s) may be the chief complaint of MC. For that reason, leptomeningeal carcinomatosis should be considered in any patient with ocular symptoms in the absence of an intraocular cause, particularly in the setting of disseminated cancer.

### Consent

Written informed consent was obtained from the patient for publication of this case report and any accompanying images. A copy of the written consent is available for review by the Editor-in-Chief of this journal.

## Competing interests

The author(s) declare that they have no competing interests.

## Authors’ contributions

The work presented here was carried out in collaboration between all authors. ALS and LMS were the major contributors in writing the manuscript. AV took and edited the images. All authors read and approved the final manuscript.

## Pre-publication history

The pre-publication history for this paper can be accessed here:

http://www.biomedcentral.com/1471-2415/12/65/prepub
